# *Drosophila* MICOS knockdown impairs mitochondrial structure and function and promotes mitophagy in muscle tissue

**DOI:** 10.1242/bio.054262

**Published:** 2020-12-03

**Authors:** Li-jie Wang, Tian Hsu, Hsiang-ling Lin, Chi-yu Fu

**Affiliations:** Institute of Cellular and Organismic Biology, Academia Sinica, Taipei 115, Taiwan

**Keywords:** *Drosophila*, MICOS, Mitochondria

## Abstract

The mitochondrial contact site and cristae organizing system (MICOS) is a multi-protein interaction hub that helps define mitochondrial ultrastructure. While the functional importance of MICOS is mostly characterized in yeast and mammalian cells in culture, the contributions of MICOS to tissue homeostasis *in vivo* remain further elucidation. In this study, we examined how knocking down expression of *Drosophila* MICOS genes affects mitochondrial function and muscle tissue homeostasis. We found that CG5903/MIC26-MIC27 colocalizes and functions with Mitofilin/MIC60 and QIL1/MIC13 as a *Drosophila* MICOS component; knocking down expression of any of these three genes predictably altered mitochondrial morphology, causing loss of cristae junctions, and disruption of cristae packing. Furthermore, the knockdown flies exhibited low mitochondrial membrane potential, fusion/fission imbalances, increased mitophagy, and limited cell death. Reductions in climbing ability indicated deficits in muscle function. Knocking down MICOS genes also caused reduced mtDNA content and fragmented mitochondrial nucleoid structure in *Drosophila*. Together, our data demonstrate an essential role of *Drosophila* MICOS in maintaining proper homeostasis of mitochondrial structure and function to promote the function of muscle tissue.

## Abstract (precis)

**Summary:** *Drosophila* MICOS knockdown impairs mitochondrial structure and function and promotes mitophagy in muscle tissue.

## INTRODUCTION

Mitochondria have a unique architecture that is required for essential cellular processes (Cogliati et al., 2016; Mannella, 2006; Zick et al., 2009). The organelles are physically separated into the matrix and intermembrane space by three membrane domains, including cristae, the inner boundary membrane (IBM), and the outer membrane; all of these mitochondrial subdomains play specific and interrelated roles in mitochondrial function. For example, the cristae harbor electron transport chain (ETC) assemblies that generate the proton gradient and membrane potential required for ATP production. As such, structural alterations in the cristae are often associated with mitochondrial dysfunction (Cogliati et al., 2016; Mannella, 2006; Zick et al., 2009). Cristae contact the IBM at cristae junctions, which require the mitochondrial contact site and cristae organizing system (MICOS) for their formation and maintenance (Harner et al., 2011; von der Malsburg et al., 2011). MICOS also interacts with outer membrane proteins and contributes to the mitochondrial intermembrane space bridging complex (Kozjak-Pavlovic, 2017; Rampelt et al., 2017; van der Laan et al., 2016; Wollweber et al., 2017). Since MICOS works as a pivot connecting different aspects of membrane architecture, the complex is considered to be essential to the biology of an integrated mitochondrion and mitochondrial network (Kozjak-Pavlovic, 2017; Rampelt et al., 2017; van der Laan et al., 2016; Wollweber et al., 2017).

The MICOS complex is formed by multiple gene products, which are not completely characterized in terms of structure and molecular interactions. In yeast, MICOS contains the MIC60 sub-complex (composed of MIC60 and MIC19) and the MIC10 sub-complex (composed of MIC10, MIC12, MIC26, and MIC27) (Kozjak-Pavlovic, 2017; Rampelt et al., 2017; van der Laan et al., 2016; Wollweber et al., 2017). In humans, the MIC60 sub-complex consists of MIC60, MIC19, and MIC25, and the MIC10 sub-complex consists of MIC10, QIL1/MIC13, MIC26, and MIC27 (Kozjak-Pavlovic, 2017; Rampelt et al., 2017; van der Laan et al., 2016; Wollweber et al., 2017). Loss of individual MICOS components causes the loss of cristae junctions and impairs complex assembly to various degrees.

Even though MICOS has been mostly characterized in yeast and mammalian cell culture, it remains to be shown how this essential complex influences tissue homeostasis. In *Drosophila*, MICOS components are less well characterized compared to those in cellular model systems. Nevertheless, *Mitofilin* (*Dmel\CG6455*) is known to be the *MIC60* homolog; its loss disrupts cristae morphology and mitochondrial motility, which leads to impaired synaptic function at neuromuscular junctions (Tsai et al., 2017). *QIL1/MIC13* (*Dmel\*CG7603), which was first identified in humans and is somewhat similar to yeast MIC12, was shown to regulate cristae morphology and cause mitochondrial network fragmentation in knockdown flies (Guarani et al., 2015; Huynen et al., 2016). In this study, we further examine the impacts of *Drosophila Mitofilin/MIC60* and *QIL1/MIC13* knockdown on mitochondrial homeostasis and tissue health. We also investigate the function of a previously uncharacterized gene, *Dmel\CG5903*, which shares homology with *MIC26* and *MIC27* based on the InterPro family and domain database (IPR019166).

We found that knocking down *CG5903/MIC26-MIC27* caused flight muscle phenotypes similar to those seen in *Mitofilin/MIC60*- and *QIL1/MIC13*-knockdown flies, including altered cristae morphology, reduced membrane potential, and increased mitochondrial network fragmentation. The knockdown of these individual genes also led to reductions in mtDNA content and fragmentation of mitochondrial nucleoids. Furthermore, the induction of mitophagy contributed to tissue homeostasis with limited cell death. In addition, we found that CG5903/MIC26-MIC27 protein has similar mitochondrial localization to Mitofilin/MIC60 and QIL1/MIC13 proteins, and it functions as a component of *Drosophila* MICOS. In summary, our study shows that *Drosophila* MICOS plays an important role in supporting mitochondria structure and network function that contribute to cellular homeostasis in muscle tissue.

## RESULTS

### Knockdown of *CG5903/MIC26-MIC27*, *Mitofilin/MIC60,* or *QIL1/MIC13* perturbs mitochondrial structure, membrane potential, and the mitochondrial network in *Drosophila* muscle tissue

**Fig. 1.**
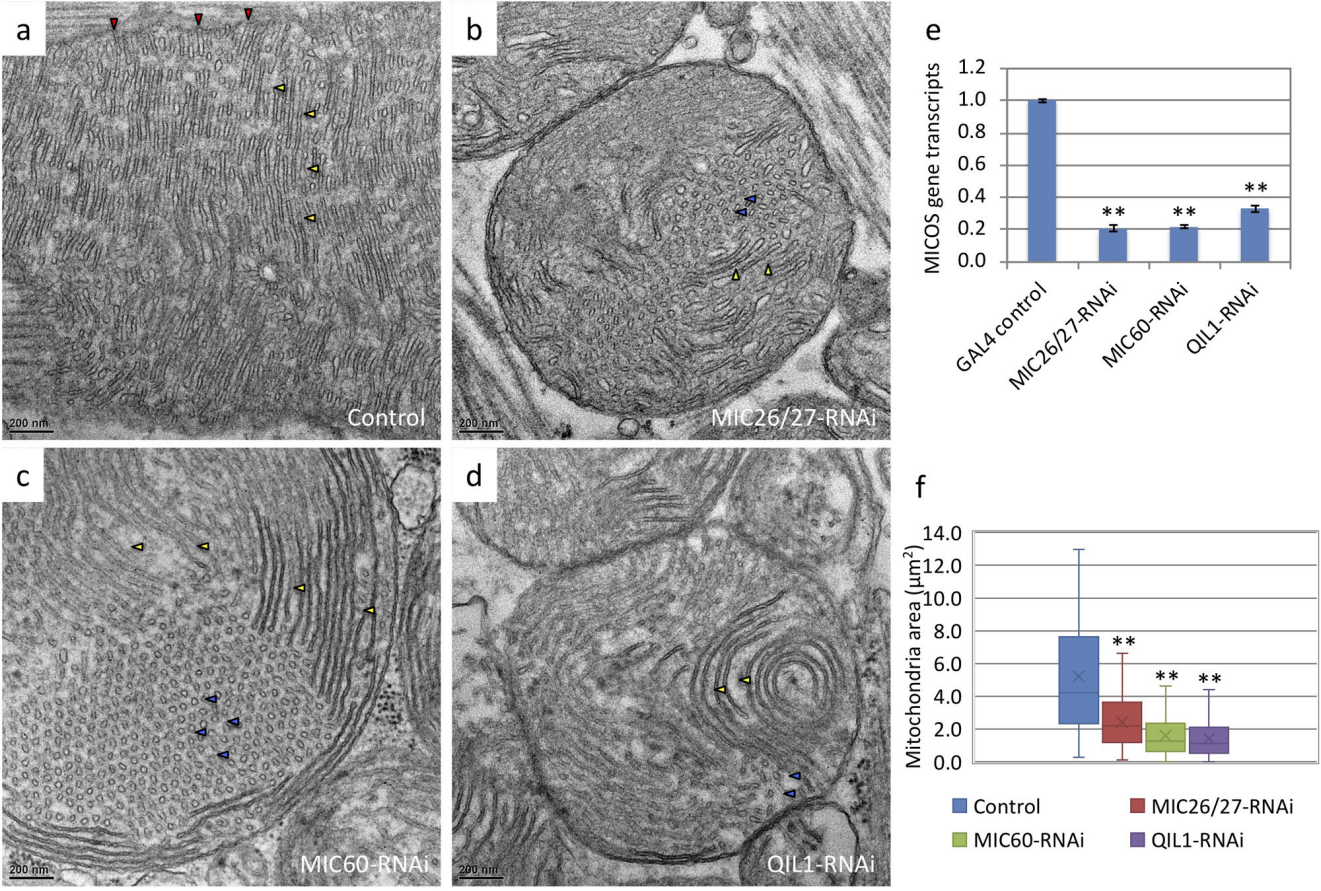
***CG5903/MIC26-MIC27*-, *Mitofilin/MIC60*-, and *QIL1/MIC13*-knockdown disrupt the mitochondrial structure and network balance.** (A–D) Thin-section EM images of *Drosophila* IFM from control, *CG5903/MIC26-MIC27*-, *Mitofilin/MIC60*-, and *QIL1/MIC13*-knockdown flies, respectively. (E) The transcript levels of *CG5903/MIC26-MIC27*-, *Mitofilin/MIC60*-, and *QIL1/MIC13* genes of the knockdown fly relative to the control assayed by qPCR. (F) The mitochondrial size distribution of knockdown flies relative to the control. (*n*=113, 193, 255, and 171 of mitochondria of the control, *CG5903/MIC26-MIC27*-, *Mitofilin/MIC60*-, and *QIL1/MIC13*-knockdown were analyzed, respectively). (Red triangle: cristae junction; yellow triangle: crista parallel to the image plane; blue triangle: crista perpendicular to the image plane; **P< 0.01). The flies of [*w, Actin88F-GAL4, CG5903/MIC26-MIC27- RNAi*], [*w, Actin88F-GAL4, Milton/MIC60- RNAi*], and [*w, Actin88F-GAL4; QIL1/MIC13- RNAi*] were used.

To explore how MICOS influences mitochondria and tissue function, we examined the phenotypes of *CG5903/MIC26-MIC27*-, *Mitofilin/MIC60*-, and *QIL1/MIC13*-knockdown flies. Good knockdown efficiency in all three lines of RNAi flies were achieved; compared to controls, respective transcript levels were 21% after *CG5903/MIC26-MIC27* knockdown, 22% after *Mitofilin/MIC60* knockdown, and 33% after *QIL1/MIC13* knockdown (Fig. 1E).

Mitochondrial ultrastructure in the knockdown flies was analyzed by thin-section transmission electron microscopy (TEM). In the control flies, mitochondria in the indirect flight muscle (IFM) contained lamellar cristae that were arranged with mostly parallel packing (Fig. 1A). The cristae junctions connecting cristae with the IBM were easily identified (Fig. 1A). On the contrary, mitochondrial ultrastructure of the *CG5903/MIC26-MIC27*-, *Mitofilin/MIC60*-, and *QIL1/MIC13*-knockdown flies was disrupted. Cristae junctions were reduced and cristae were detached from the IBM (Fig. 1B–D). The cristae packing also appeared to be defective, as cristae were arranged in multiple directions. The disruption of cristae directionality in the MICOS-knockdown mitochondria suggests that cristae junctions might function as anchor points to align cristae. *CG5903/MIC26-MIC27*-knockdown showed similar disruption of mitochondrial morphology as *Mitofilin/MIC60*-, and *QIL1/MIC13*-knockdowns.

**Fig. 2.**
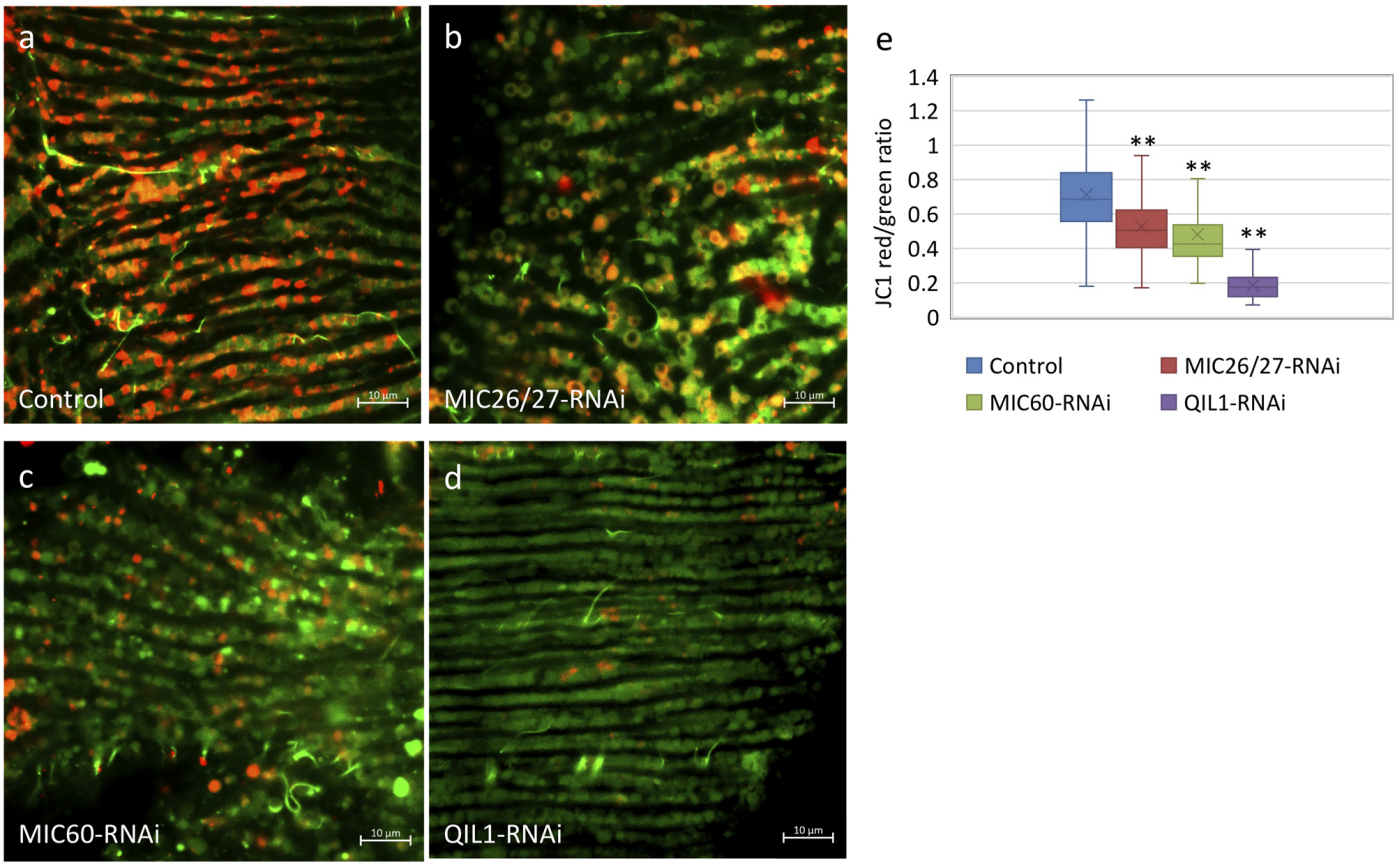
***CG5903/MIC26-MIC27*-, *Mitofilin/MIC60*-, and *QIL1/MIC13*-knockdown flies exhibit low mitochondrial membrane potential.** (a–d) JC1 staining of IFM from *CG5903/MIC26-MIC27*-, *Mitofilin/MIC60*-, and *QIL1/MIC13*-knockdown flies. Red fluorescence marks high mitochondrial membrane potential, whereas green fluorescence indicates low mitochondrial membrane potential. (e) The ratio of red to green fluorescent intensity was analyzed and plotted. (Triplicates of a volume of 84.2×84.2×5 μm^3^ of were analyzed). **P<0.01. The flies of [*w, Actin88F-GAL4, CG5903/MIC26-MIC27- RNAi*], [*w, Actin88F-GAL4, Milton/MIC60- RNAi*], and [*w, Actin88F-GAL4; QIL1/MIC13- RNAi*] were used.

In addition to alterations in mitochondrial ultrastructure, smaller sized mitochondria were more populous in *CG5903/MIC26-MIC27*-, *Mitofilin/MIC60*-, and *QIL1/MIC13*-knockdown flies (Fig. S1a–d). Analysis of mitochondrial sizes from thin-section TEM images, the median of the mitochondrial size distribution was 52%, 30%, and 27% smaller in the *CG5903/MIC26-MIC27*-, *Mitofilin/MIC60*-, and *QIL1/MIC13*-knockdown lines comparing to the control, respectively (Fig. 1F). The balance of fission and fusion was shifted toward fission in all three MICOS knockdown lines. Similar fragmentation of the mitochondrial network was also reported in the previous study on *QIL1/MIC13*-knockdown flies (Guarani et al., 2015). Because the mitochondrial network was fragmented and mitochondrial dynamics often correlate with function, we next examined mitochondrial membrane potential. Staining with the membrane potential-sensitive dye, JC-1, revealed the mitochondria in IFM of *CG5903/MIC26-MIC27*-, *Mitofilin/MIC60*-, and *QIL1/MIC13*-knockdown flies exhibited lower membrane potential than the controls, reflecting by the lower ratio of red versus green fluorescent intensity (Fig. 2A–E). The median of the ratio in the analyzed pools was 0.7-, 0.6-, and 0.3-fold in the *CG5903/MIC26-MIC27*-, *Mitofilin/MIC60*-, and *QIL1/MIC13*-knockdown flies than in the control flies, where triplicates of volumes of 84.2×84.2×5 μm^3^ were analyzed (Fig. 2E).

In summary, *CG5903/MIC26-MIC27*-knockdown mitochondria had a similar phenotype as those observed in *Mitofilin/MIC60*- and *QIL1/MIC13*-knockdowns, with mitochondria lacking cristae junctions and exhibiting non-uniform cristae directionality. The detached and disorganized cristae structure was coincident with low mitochondrial membrane potential and altered fusion/fission balance in the mitochondrial network.

### *CG5903/MIC26-MIC27*, *Mitofilin/MIC60*, and *QIL1/MIC13*-knockdown flies have increased mitophagy but limited cell death

**Fig. 3.**
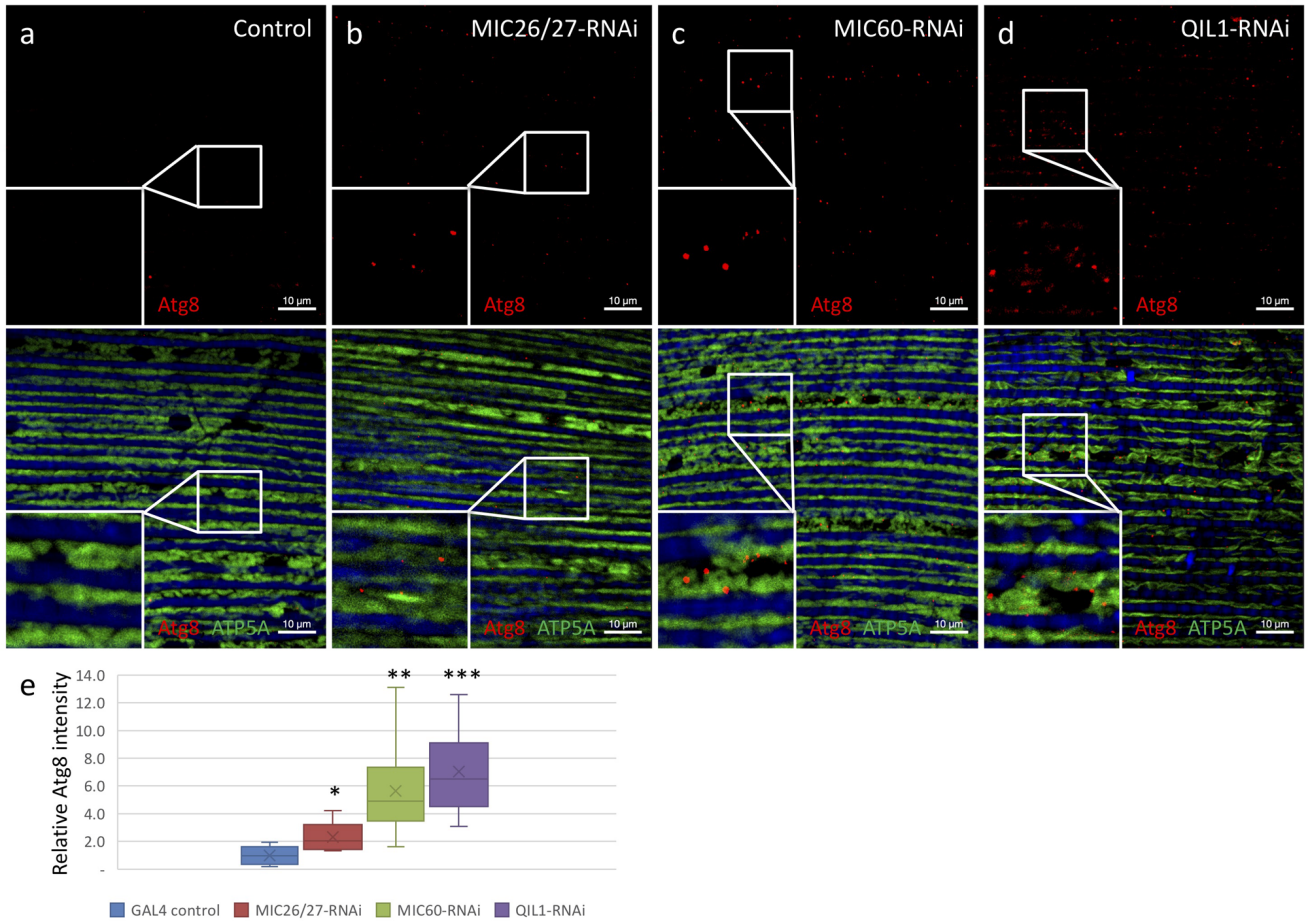
***CG5903/MIC26-MIC27*-, *Mitofilin/MIC60*-, and *QIL1/MIC13*-knockdown flies have increased mitophagy.** (a–d) IFMs of *CG5903/MIC26-MIC27*-, *Mitofilin/MIC60*-, and *QIL1/MIC13*-knockdown flies were stained for autophagy marker Atg8 (red), ATP5A (mitochondria in green), and phalloidin (muscle fibers in blue) showing increased mitophagy of the knockdown flies. (e) The Atg8 fluorescent intensities of the knockdown flies were analyzed and compared to the control flies. (Nice-replicates of a volume of 84.2×84.2×5 μm^3^ of were analyzed). *P<0.05; **P< 0.01; ***P<0.001. The flies of [*w, Actin88F-GAL4, CG5903/MIC26-MIC27- RNAi*], [*w, Actin88F-GAL4, Milton/MIC60- RNAi*], and [*w, Actin88F-GAL4; QIL1/MIC13- RNAi*] were used.

**Fig. 4.**
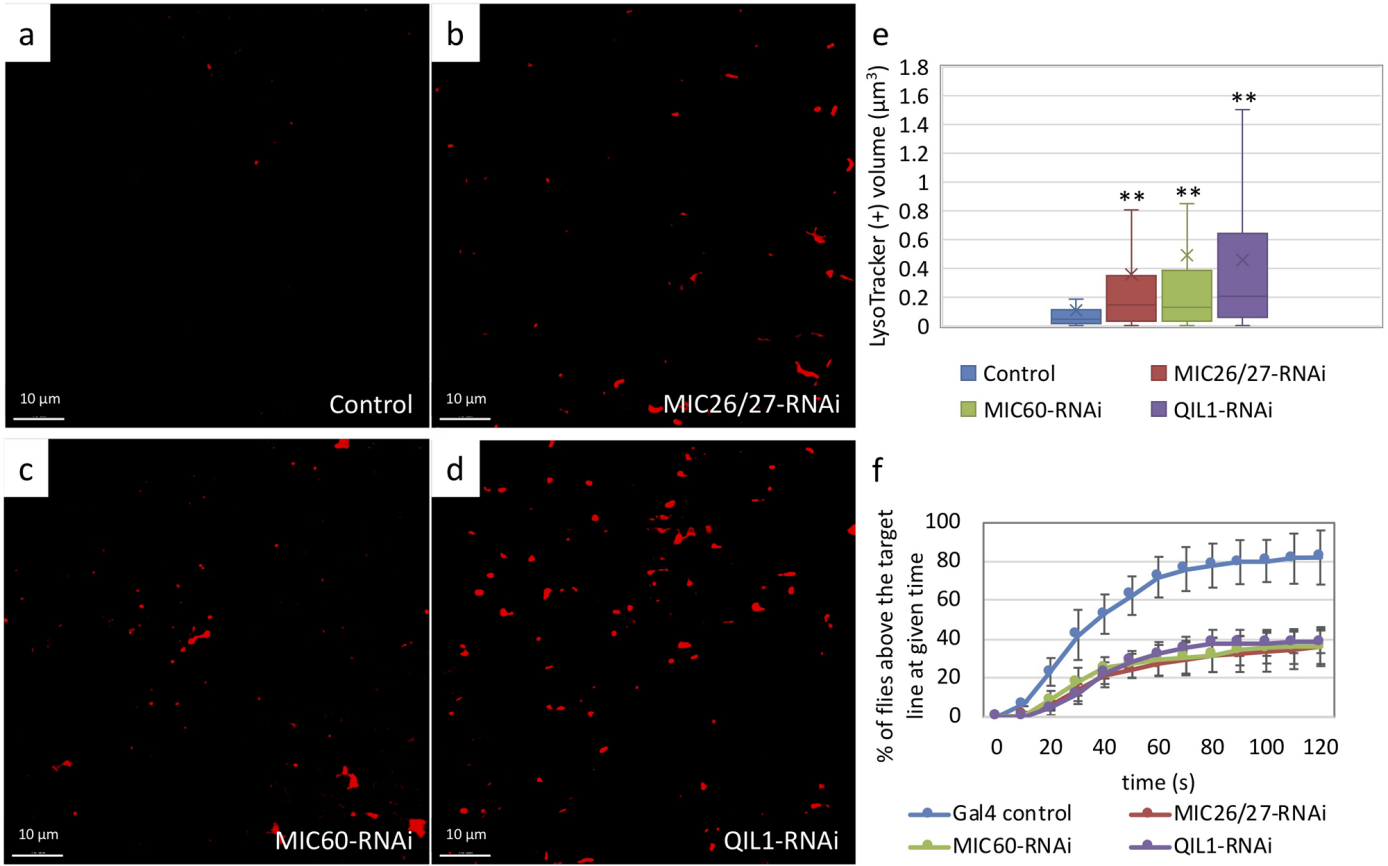
***CG5903/MIC26-MIC27*-, *Mitofilin/MIC60*-, and *QIL1/MIC13*-knockdown flies have compromised climbing ability and increased lysosomal degradation.** (a–d) LysoTracker staining of IFM from *CG5903/MIC26-MIC27*-, *Mitofilin/MIC60*-, and *QIL1/MIC13*-knockdown flies. Red fluorescence marks acidic lysosomal compartments. (e) The red fluorescent intensities of the knockdown flies were analyzed and compared to the control flies. (Triplicates of a volume of 84.2×84.2×5 μm^3^ of were analyzed). **P<0.01. (f) Fly climbing ability was analyzed over 120 s. *CG5903/MIC26-MIC27*-, *Mitofilin/MIC60*-, and *QIL1/MIC13*-knockdown flies showed compromised climbing ability compared to controls. (*n*=56, 69, 79, and 62 of the control, *CG5903/MIC26-MIC27*-, *Mitofilin/MIC60*-, and *QIL1/MIC13*-knockdown flies, respectively, were used in the analysis.) The flies of [*w, Actin88F-GAL4, CG5903/MIC26-MIC27- RNAi*], [*w, Actin88F-GAL4, Milton/MIC60- RNAi*], and [*w, Actin88F-GAL4; QIL1/MIC13- RNAi*] were used.

**Fig. 5.**
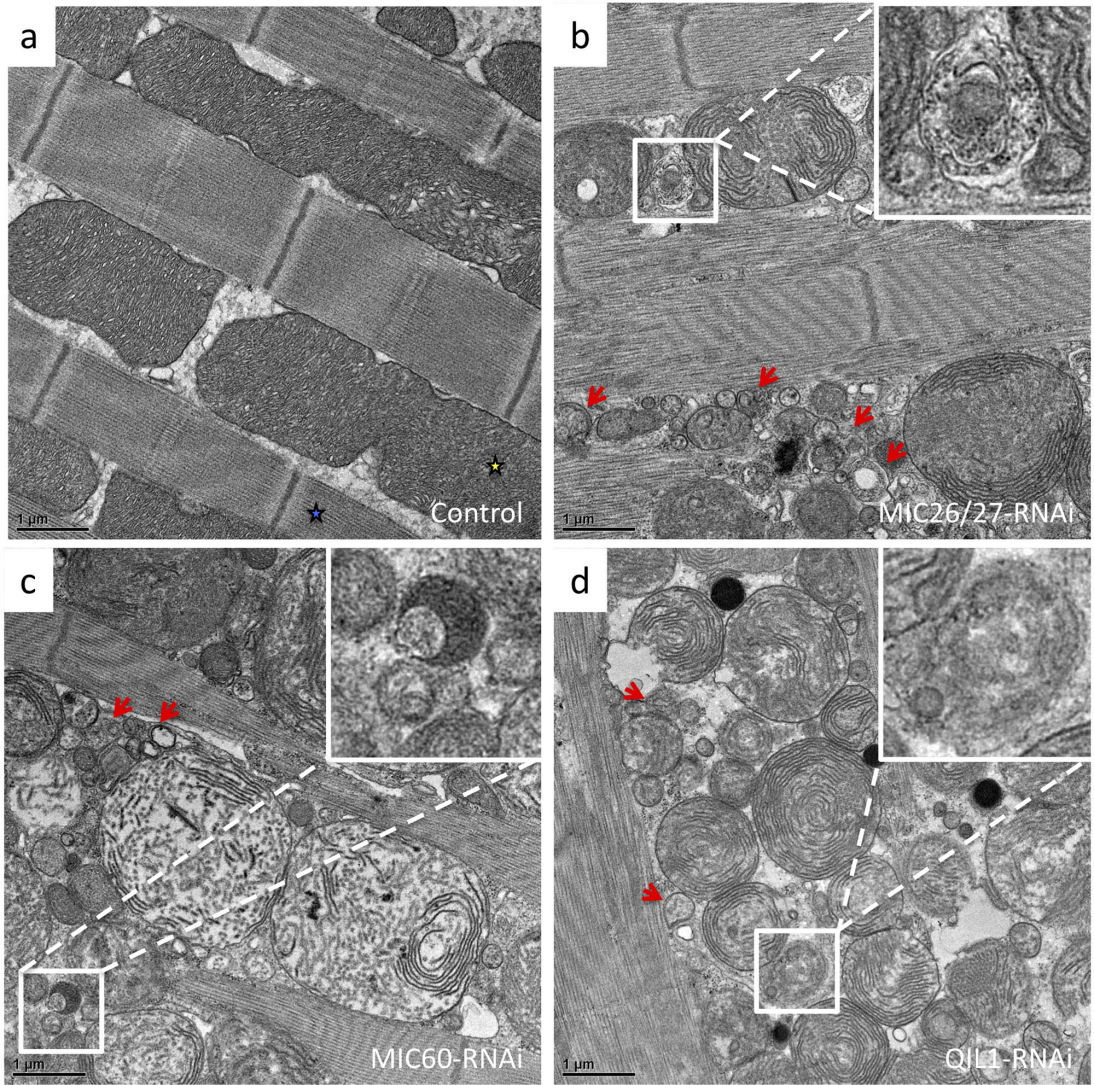
***CG5903/MIC26-MIC27*-, *Mitofilin/MIC60*-, and *QIL1/MIC13*-knockdown flies exhibit upregulated mitophagy.** (a–d) Thin-section EM images of *Drosophila* IFM from control, *CG5903/MIC26-MIC27*-, *Mitofilin/MIC60*-, and *QIL1/MIC13*-knockdown flies, showing the mitophagic structures (red arrows). The flies of [*w, Actin88F-GAL4, CG5903/MIC26-MIC27- RNAi*], [*w, Actin88F-GAL4, Milton/MIC60- RNAi*], and [*w, Actin88F-GAL4; QIL1/MIC13- RNAi*] were used. Yellow stars, mitochondria; blue stars, muscle fibers.

The climbing ability of all three MICOS-knockdown flies seemed to be compromised that displayed about a 45% reduction in climbing ability, even though the muscle tissue integrity was maintained according to the thin-section EM and immunofluorescence analysis (Fig. 4F; Fig. S1,2). MICOS-knockdown flies did not show muscle loss. The percent area of the muscle fibers in the IFM tissue in an imaged area of 7.1×10^3 ^μm^2^ didn't differ significantly from the control flies (Fig. S2e). We, therefore, examined whether mitophagy was promoted in MICOS-knockdown flies. The IFMs of MICOS-knockdown flies were stained with anti-Atg8 antibodies as an autophagy marker. *CG5903/MIC26-MIC27*, *Mitofilin/MIC60*, *QIL1/MIC13*-knockdown flies showed 2.3, 5.6, and 7-fold increased signals of the fluorescent intensities of Atg8, respectively (Fig. 3A-E). In addition, the LysoTracker staining of IFM of MICOS-knockdown flies was performed in three samplings. IFM volumes of 2.9×10^4^ μm^3^ were imaged as a dataset. The volumes of positive LysoTracker signals were analyzed. *CG5903/MIC26-MIC27*-, *Mitofilin/MIC60*-, *QIL1/MIC13*-knockdown flies showed 3.2-, 2.8-, 4.5-fold increased signals of acidic lysosomal compartments than the control, respectively, where triplicates of volumes of 84.2×84.2×5 μm^3^ were analyzed (Fig. 4A–E; Fig. S3). In line with the observation, mitophagic structures were frequently identified in the thin-section TEM micrographs of IFM from MICOS-knockdown flies (Fig. 5A–D).

**Fig. 6.**
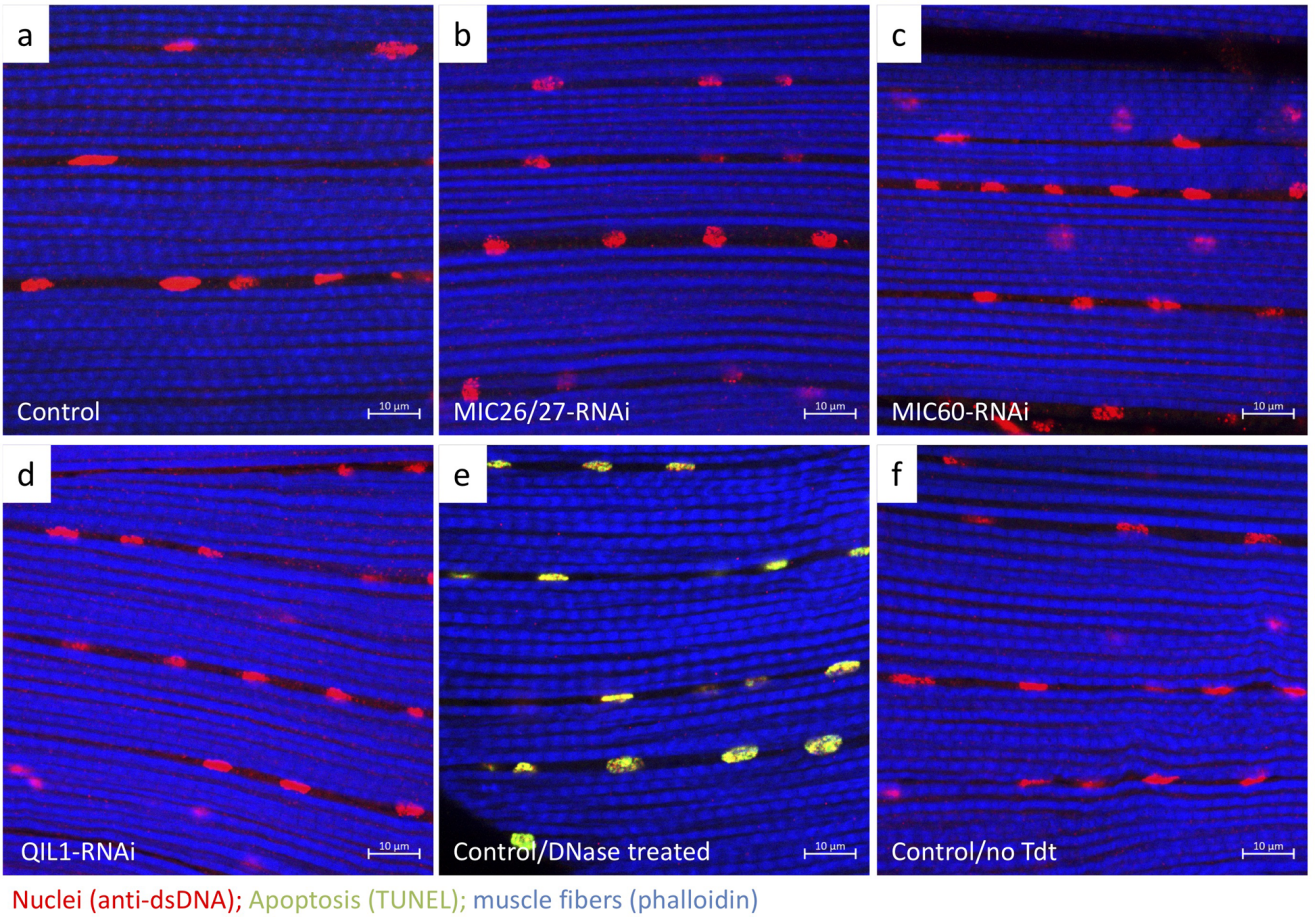
***CG5903/MIC26-MIC27-*, *Mitofilin/MIC60*-, and *QIL1/MIC13*-knockdown flies do not have elevated apoptosis.** (a–d) TUNEL staining of *Drosophila* IFM from control, *CG5903/MIC26-MIC27*-, *Mitofilin/MIC60*-, and *QIL1/MIC13*-knockdown flies. The positive and negative controls for the TUNEL assay using the control flies were shown in (e) and (f), respectively. (Volumes of 84.2×84.2×5 μm^3^ were analyzed). Positive TUNEL signals are shown in green; nuclei were stained with anti-dsDNA, red; muscle fibers were stained by phalloidin, purple. The flies of [*w, Actin88F-GAL4, CG5903/MIC26-MIC27- RNAi*], [*w, Actin88F-GAL4, Milton/MIC60- RNAi*], and [*w, Actin88F-GAL4; QIL1/MIC13- RNAi*] were used.

On the other hand, apoptosis was not elevated in MICOS-knockdown flies. The TUNEL assay was applied to detect apoptotic cells by labeling DNA strand breaks. The *CG5903/MIC26-MIC27-*, *Mitofilin/MIC60-*, and *QIL1/MIC13*-knockdown IFM tissues showed low levels of apoptotic nuclei (less than 1%), which were comparable to the levels in the control (Fig. 6A–D; Fig. S4). Of note, the positive control with DNase I digestion showed 100% nuclei with positive signals (Fig. 6E; Fig. S4). Triplicates of volumes of 84.2×84.2×5 μm^3^ were analyzed in the assay. These data suggest that mitophagy was enhanced to degrade dysfunctional MICOS-knockdown mitochondria, and this quality control appears to be sufficient to maintain muscle tissue integrity and prevent excessive apoptosis.

### *CG5903/MIC26-MIC27-*, *Mitofilin/MIC60-* and *QIL1/MIC13*-knockdown flies display reduced mtDNA content and fragmented mitochondrial nucleoids

**Fig. 7.**
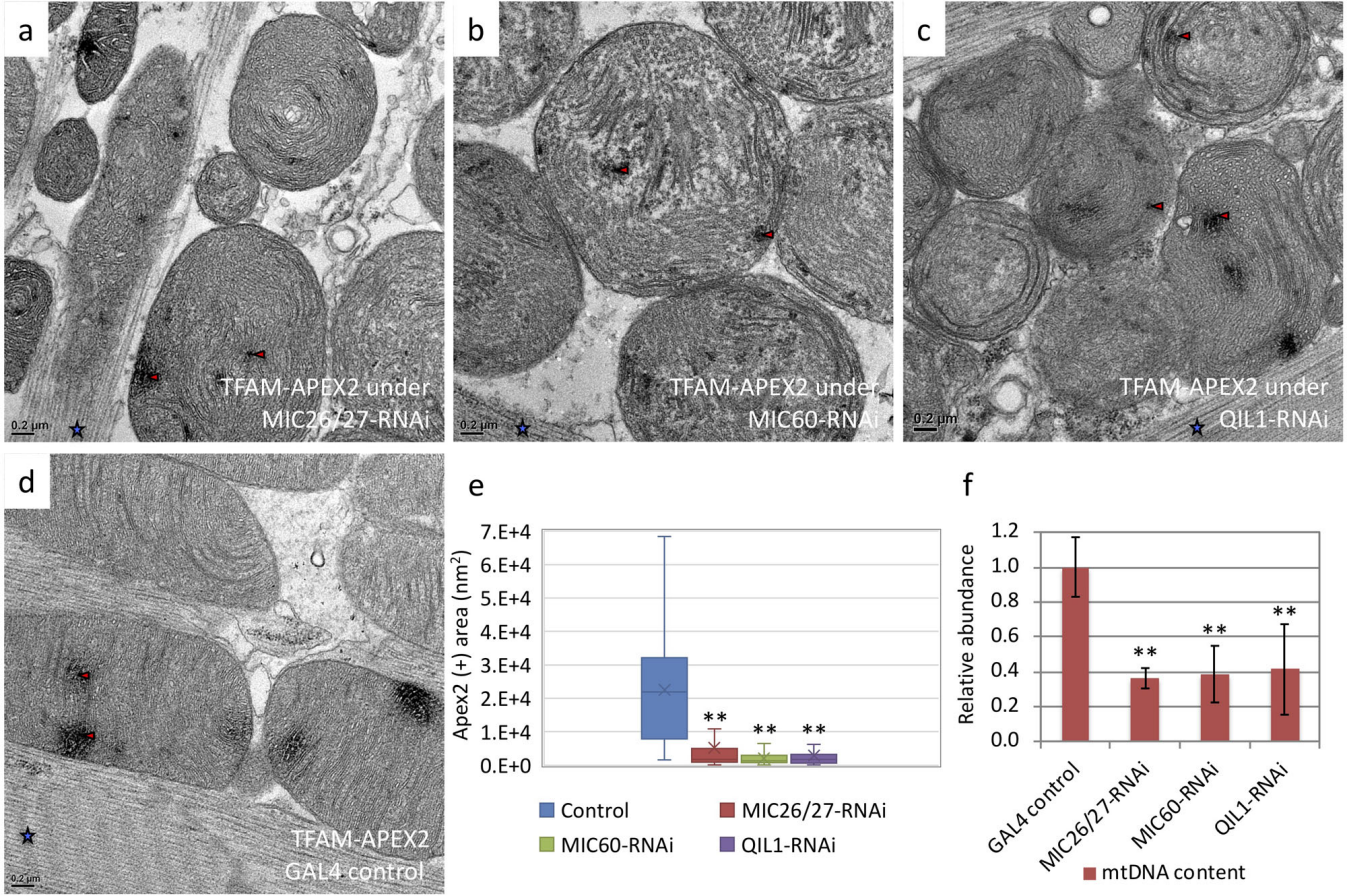
***CG5903/MIC26-MIC27*-, *Mitofilin/MIC60*-, and *QIL1/MIC13*-knockdown flies had reduced mtDNA content and fragmented mitochondrial nucleoid organization.** (a–d) Apex2-EM images of TFAM-Apex2 flies with knockdown of *CG5903/MIC26-MIC27*, *Mitofilin/MIC60*, and *QIL1/MIC13* genes. (e) The size distributions of mitochondrial nucleoids were analyzed based on TFAM-Apex2 staining. (*n*=44, 204, 167, and 94 of mitochondria of the control, *CG5903/MIC26-MIC27*-, *Mitofilin/MIC60*-, and *QIL1/MIC13*-knockdown were analyzed, respectively). (f) Relative mtDNA content was determined by qPCR. Red triangle, positive Apex2 staining; **P<0.01. The flies of [*w, Actin88F-GAL4, CG5903/MIC26-MIC27- RNAi*], [*w, Actin88F-GAL4, Milton/MIC60- RNAi*], and [*w, Actin88F-GAL4; QIL1/MIC13- RNAi*] were used. Blue stars, muscle fibers.

Mitochondrial DNA encodes proteins that are essential for oxidative respiratory function. Therefore, its stability and integrity are associated with mitochondrial function (Bogenhagen et al., 2008; Kang et al., 2018; Nicholls and Gustafsson, 2018). To investigate how knockdown of *CG5903/MIC26-MIC27*, *Mitofilin/MIC60*, and *QIL1/MIC13* affect mtDNA content, we monitored the mtDNA copy number by qPCR. The level of an mtDNA gene, mitochondrial Cytochrome c oxidase subunit III (COIII), was normalized to that of the nuclear gene, Ribosomal protein L32 (RpL32). Relative to controls, *CG5903/MIC26-MIC27-*, *Mitofilin/MIC60-* and *QIL1/MIC13*-knockdown flies only carried 36%, 39% and 41% mtDNA, respectively (Fig. 7F). Therefore, *Drosophila* MICOS genes are required for the maintenance of mtDNA levels.

mtDNA is arranged in nucleoid-like structures (mtNucleoid) that associate with a variety of proteins important for mtDNA stability, replication, and transcription (Lee and Han, 2017; Ngo et al., 2014; Takamatsu et al., 2002). Among the mtNucleoid-associated proteins, transcription factor A (TFAM) functions as a major structural protein that binds and packages mtDNA independent of the nucleotide sequence, in addition to its role as a transcription factor (Lee and Han, 2017; Ngo et al., 2014; Takamatsu et al., 2002). Since TFAM exists predominantly in an mtDNA-bound state, it has been widely used as a marker to image mtNucleoids by EM or fluorescence microscopy (Brown et al., 2011; Han et al., 2017; Kopek et al., 2012; Kukat et al., 2011; McArthur et al., 2018). To investigate whether MICOS knockdown affects mtNucleoid organization, we applied an *in situ* staining method for Apex2 in TFAM-Apex2 knock-in flies with concurrent knockdown of MICOS genes. Apex2 is an ascorbate peroxidase that catalyzes the polymerization of DAB in the presence of hydrogen peroxide (H_2_O_2_). Polymerized DAB enhances EM contrast after osmium tetraoxide staining and allows protein localization to be tracked with ultrastructural resolution (Martell et al., 2012). The Apex2 tag was fused to the c-terminus of endogenous TFAM to minimize possible confounding effects on the TFAM expression level, and the TFAM-Apex2 knock-in fly is homozygous viable, which suggests the TFAM-Apex2 fusion protein can substitute for wild-type TFAM protein.

The expression of TFAM-Apex2 was analyzed by western blot in *CG5903/MIC26-MIC27-*, *Mitofilin/MIC60-*, and *QIL1/MIC13-*knockdown flies and showed similar levels of expression in all three lines and the control (Fig. S5b). IFM was then subjected to Apx2-EM analysis, in which TFAM-Apex2 signals appear as dark staining in the EM micrographs (Fig. 7A–D). The TFAM-Apex2 GAL4 control had normal mtNucleoid organization compared to the TFAM-Apex2 control flies (Fig. 7D, Fig. S5a). In the MICOS-knockdown lines, major reductions in the size of mitochondrial nucleoids were observed, even though TFAM-Apex2 protein expression remained similar to controls (Fig. 7A–D, Fig. S5b). According to our analysis of EM micrographs, TFAM-Apex2 densities in the respective mitochondria populations in *CG5903/MIC26-MIC27-*, *Mitofilin/MIC60*- and *QIL1/MIC13*-knockdown muscle were 4%, 3%, and 3% of the control, where 44, 204, 167, and 94 mitochondria were analyzed, respectively (Fig. 7E).

In conclusion, *CG5903/MIC26-MIC27*-, *Mitofilin/MIC60*-, and *QIL1/MIC13*-knockdown impair mtDNA maintenance and the stability and integrity of mtNucleoids.

### CG5903/MIC26-MIC27, Mitofilin/MIC60, and QIL1/MIC13 are all localized to the cristae junction, nearby the IBM, and extended cristae

To characterize the sub-mitochondrial localization of CG5903/MIC26-MIC27 protein and compare it to that of Mitofilin/MIC60 and QIL1*/MIC13*, we utilized the Apex2 EM labeling method to track protein localization at ultrastructural resolution. We generated expression constructs with Apex2 tags fused to the c-termini of *CG5903/MIC26-MIC27*, *Mitofilin/MIC60*, and *QIL1/MIC13* genes.

**Fig. 8.**
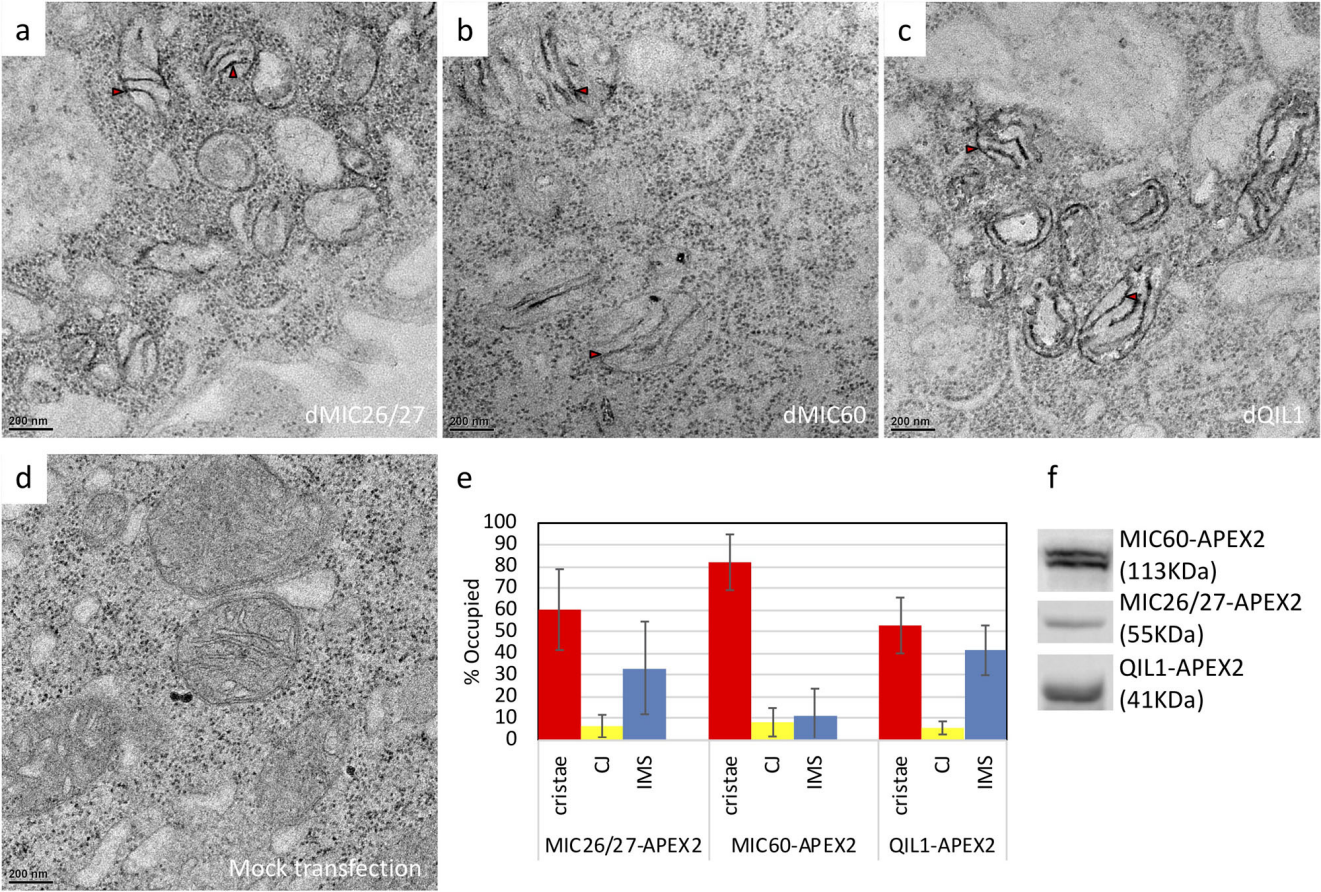
**CG5903/MIC26-MIC27, MIC60, and QIL1/MIC13 localized to cristae junctions, the nearby IBM, and extended cristae.** (a–c) Apex2-EM staining of S2 cells transfected with plasmids expressing *Drosophila* *melanogaster* CG5903/MIC26-MIC27, MIC60, and QIL1/MIC13-Apex2 fusion proteins. (d) Negative control Apex2-EM staining of mock-transfected cells. (e) Mitochondrial ultrastructure distribution of CG5903/MIC26-MIC27, MIC60, and QIL1-Apex2 fusion proteins. (*n*=11, 17, and 15 of mitochondria of *CG5903/MIC26-MIC27*-, *Mitofilin/MIC60*-, and *QIL1/MIC13*-knockdown were analyzed, respectively). (f) Western blot analysis of the expression of CG5903/MIC26-MIC27, MIC60, and QIL1/MIC13-Apex2 fusion proteins. Red triangle, positive Apex2 staining. The flies of [*w, Actin88F-GAL4, CG5903/MIC26-MIC27- RNAi*], [*w, Actin88F-GAL4, Milton/MIC60- RNAi*], and [*w, Actin88F-GAL4; QIL1/MIC13- RNAi*] were used.

The expression of the MICSO-Apex2 fusion proteins in S2 cells was confirmed by western blot (Fig. 8F). The cells were subjected to Apex2 EM staining and thin-section EM analysis. Apex2-fusion proteins appeared as darkly stained areas in the EM micrographs. The EM images showed CG5903/MIC26-MIC27-Apex2, Mitofilin/MIC60-Apex2, and QIL1/MIC13-Apex2 staining all appeared specifically in the mitochondria, indicating correct targeting of the fusion constructs. Mock-transfected cells showed no enhanced contrast, as a negative control for Apex2 staining (Fig. 8D). Moreover, CG5903/MIC26-MIC27, Mitofilin/MIC60, and QIL1/MIC13 proteins showed similar localization within the mitochondria (Fig. 8A-C). Each protein appeared at the cristae junction and also in the nearby IBM and extended cristae. Previous studies using Apex2 EM staining of human MIC60 and MIC19 also reported similar mitochondrial localization patterns (Sastri et al., 2017). Also, in line with our observations, a previous study showed immunostaining of MIC60 produced signals at cristae junctions and within the nearby IBM and cristae to a minor degree (Jans et al., 2013). In conclusion, CG5903/MIC26-MIC27 had a similar pattern of mitochondrial localization as Mitofilin/MIC60 and QIL1/MIC13 proteins, which are known to function as *Drosophila* MICOS components.

## DISCUSSION

In this study, we characterized the role of *Drosophila* MICOS genes in maintaining mitochondria and muscle function. We compared the function of *Drosophila CG5903/MIC26-MIC27*, a homolog of *MIC26* and *MIC27*, to two known MICOS components, *Mitofilin/MIC60* and *QIL1/MIC13*. Our data showed similar phenotypes for *CG5903/MIC26-MIC27-*, *Mitofilin/MIC60-*, and *QIL1/MIC13*-knockdown flies. In all the knockdown strains, altered mitochondrial morphology was observed, including the loss of cristae junctions and non-uniformity in the cristae packing orientation. In addition, low mitochondrial membrane potential was observed in knockdown mitochondria along with altered mitochondrial fusion/fission balance that resulted in reduced mitochondrial size and fragmented networks. Furthermore, mitophagy was enhanced, presumably to degrade dysfunctional mitochondria and prevent cell death in the knockdown IFM tissue. *Drosophila* MICOS knockdowns also exhibited loss of mtDNA content and fragmented mitochondrial nucleoid structures. Together, these results suggest that *Drosophila* MICOS is essential for mitochondrial function, mtDNA maintenance, and muscle function in the IFM.

MICOS is a sophisticated multi-protein assembly consisting of the MIC60 sub-complex and the MIC10 sub-complex (Kozjak-Pavlovic, 2017; Rampelt et al., 2017; van der Laan et al., 2016; Wollweber et al., 2017). Previous studies showed a lack of MIC60 and MIC10 causes more pronounced phenotypes than the deficiency of other MICOS components. MIC60 is one of the core components, which mediates the interaction of MICOS with the mitochondrial inter-membrane space assembly (MIA) as well as proteins in the outer membrane, including sorting and assembly machinery (SAM) and translocase of the outer membrane (TOM) (Kozjak-Pavlovic, 2017; Rampelt et al., 2017; van der Laan et al., 2016; Wollweber et al., 2017). MIC60 knockdown was previously shown to affect mtDNA integrity in human cells and yeast, and our study shows the same is true in *Drosophila* (Itoh et al., 2013; Li et al., 2016; Rossi et al., 2009; Yang et al., 2015). In mammalian cells, downregulation of MIC60 induces the formation of giant mitochondria accompanied by the appearance of clustered mitochondrial nucleoids and reduced mtDNA transcription (Li et al., 2016; Yang et al., 2015). Furthermore, yeast lacking MIC60 has a reduced number of large mitochondrial nucleoids (Itoh et al., 2013). In *Drosophila*, we observed reduced mtDNA content and mtNucleoids of relatively small size.

QIL1/MIC13, which is related to yeast MIC12, stabilizes the MIC10 sub-complex and mediates its interaction with the MIC60 sub-complex to form a mature MICOS complex (Guarani et al., 2015; Huynen et al., 2016). MIC26 and MIC27 belong to the apolipoprotein O family and function as a part of the MIC10 sub-complex, with MIC27 stabilizing MIC10 oligomers (Kozjak-Pavlovic, 2017; Rampelt et al., 2017; van der Laan et al., 2016; Wollweber et al., 2017). The deletion of MIC27 in yeast results in more pronounced cristae structure defects than the deletion of MIC26 (Kozjak-Pavlovic, 2017; Rampelt et al., 2017; van der Laan et al., 2016; Wollweber et al., 2017). Knockdown of *Drosophila CG5903/MIC26-MIC27* alters cristae architecture, mtDNA integrity, and mitochondrial network function, similar to the phenotypes of other MICOS gene knockdowns.

*Drosophila* CG5903/MIC26-MIC27, Mitofilin/MIC60, and QIL1/MIC13 were all localized to cristae junctions, the IBM surrounding the cristae junctions, and the extended cristae, similar to the results of previous studies utilizing Apex2 labeling of human MIC60 and MIC19 or immunolabeling of MIC60 (Jans et al., 2013; Sastri et al., 2017). The restriction of MICOS localization to the IBM immediately surrounding the cristae junctions suggests that targeting of MICOS proteins is highly precise. In agreement with this idea, MICOS was shown to interact with proteins in the IBM and cristae, including OPA1 (mediates inner membrane fusion and cristae remolding) and subunit IV of cytochrome c oxidase; by these interactions, MICOS can coordinate ETC function (Friedman et al., 2015; Harner et al., 2014; Hoppins et al., 2011; Schweppe et al., 2017). Super-resolution fluorescence microscopy studies also showed MIC60 exists in a clustered distribution as a part of a multi-protein interaction network that scaffolds mitochondria (Stoldt et al., 2019).

MICOS functions as a hub of interactions that define the shape of the mitochondrial double membrane. Along with its architectural role, the functional roles of MICOS in metabolism, calcium homeostasis, and protein and lipid biogenesis are beginning to be discovered (Kozjak-Pavlovic, 2017; Rampelt et al., 2017; van der Laan et al., 2016; Wollweber et al., 2017). Among these functions, MIC60 phosphorylation by protein kinase A was shown to regulate PINK1 stability and Parkin recruitment to damaged mitochondria (Akabane et al., 2016). The stimulation of PINK1/Parkin signaling initiates mitophagy, which is an essential quality control mechanism of clearing dysfunctional mitochondria to maintain mitochondrial network function (Geisler et al., 2010; Kim et al., 2007; Youle and Narendra, 2011). Here we showed that mitophagy was upregulated in MICOS-knockdown *Drosophila* with no apparent increase in cell death. These results suggest autophagy may be sufficient to prevent apoptosis and maintain tissue integrity in MICOS knockdown flies (Ji and Yeo, 2019; Kubli and Gustafsson, 2012; Morales et al., 2019; Palikaras et al., 2018). Together, our results delineate the role of *Drosophila* MICOS as a key factor in the maintenance of the mitochondrial structure and network function to enhance the function of muscle tissue.

## MATERIALS AND METHODS

### Fly strains

A *Drosophila* strain on the Oregon-R-P2 background was used as the wild type. *MICOS-RNAis* were expressed in the indirect flight muscle by *Actin88F-GAL4* (Bloomington 38459). The *UAS-RNAi* used in the study were *P{TRiP.HMS05459}attp40* (for *CG5903/MIC26-MIC27*; Bloomington 66933), *P{TRiP.HMJ30307}attp40* (for *Milton/MIC60*; Bloomington 63994), and *P{TRiP.GLC01383}attp2* (for *QIL1/MIC13*; Bloomington 44634). The genotypes of the flies are listed in Table 1.

**Table 1.**
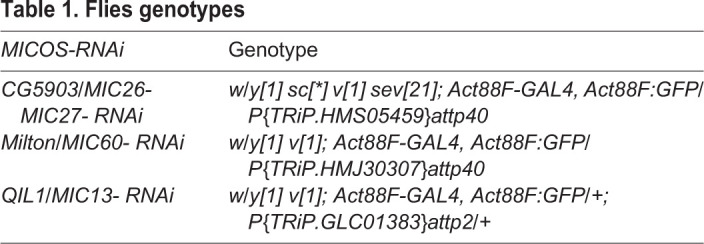
**Flies genotypes**

TFAM-APEX2 knock-in flies were generated by CRISPR/Cas9-mediated genome editing and homology-dependent repair using a guide RNA(s) and a dsDNA plasmid donor. The PBac system was used to facilitate genetic screening (Well Genetics). The construct design was detailed as follows.

Guide RNA Primers: Sense oligo 5′- CTTCGCCAAAGCCCCGCAAGACGC;

Antisense oligo 5′- AAACCGCGTCTTGCGGGGCTTTGGC

PAM mutation: GCCAAAGCCCCGCAAGACGC[TGG] CTG→CTC/ L→L

Upstream Homology Arm: 1083 bp, −1086 to −4 nt relative to stop codon of TFAM

Forward oligo 5′- TGCCAATCCCCAGATTACCAC;

Reverse oligo 5′-TATATCTTTGGAGGCGAGCGT

Downstream Homology Arm: 1026 bp, +1 to +1028 nt relative to stop codon of TFAM

Forward oligo 5′-TTGTAGCTGCTCGGCCCGC;

Reverse oligo 5′-AAATGATGCAGAAGTGGCT

TFAM-APEX2 on a *MICOS-RNAi* background was expressed in the indirect flight muscle by *Actin88F-GAL4* (Bloomington 38459).

### Thin-section TEM for morphological observation

The experiments were carried out as previously described with some modifications (Jiang et al., 2017a,b, 2020). Flies were anesthetized on ice and embedded in 4% low melting agarose in 0.1 M phosphate buffer. Embedded flies were then sectioned at 100 μm with a vibrating blade microtome (Leica VT1200S) and fixed in 2% glutaraldehyde in buffer containing 0.1 M sodium cacodylate with 2 mM CaCl_2_, pH 7 for 60 min followed by washing (2 min in the buffer, five times). The sections were post-fixed in 2% osmium tetroxide for 30 min followed by washing (2 min in the buffer, five times), after which samples were incubated in 2% uranyl acetate overnight. After dehydration in ascending percentages of ethanol, the specimens were infiltrated and embedded in Spurr's resin and polymerized at 65°C for 16 h. The specimen blocks were trimmed and sectioned using an ultramicrotome. The sections were stained with 2% uranyl acetate for 10 min, Reynold's lead citrate for 4 min, and subjected to TEM inspection.

The mitochondria size distribution was determined using Amira-Avizo 3D visualization and analysis software (Thermo Fisher Scientific), where individual mitochondria were defined manually and the areas were output and analyzed (Fig. S2a–d; Fig. 1F). The analysis showed in Fig. 1f included 113, 193, 255, and 171 of mitochondria of the control, *CG5903/MIC26-MIC27*-, *Mitofilin/MIC60*-, and *QIL1/MIC13*-knockdown in the analysis, respectively.

### Immunofluorescence staining

The experiments were carried out as previously described with some modifications (Macchi et al., 2013). Fly thoraxes were dissected into halves in fixation buffer containing 4% paraformaldehyde and 1% Triton X-100 in PBS and fixed for 20 min at room temperature (RT) without shaking. The specimens were washed with 0.1% Triton X-100 in PBS for 20 min at RT three times. After blocking with 5% normal goat serum (Jackson ImmunoResearch) in 0.1% Triton X-100 in PBS for 2 h at RT, the specimens were stained with primary mouse anti-ATP5A (1:1000, Abcam 14748) and/or primary mouse anti-GABARAP+GABARAPL1+GABARAPL2 antibody (1:500, Abcam ab109364) in blocking buffer overnight at 4°C. After washing with 0.1% Triton X-100 in PBS for 20 min at RT three times, the specimens were stained with secondary antibody (1: 1000 dilutions, anti-mouse IgG Alexa-488, Jackson ImmunoResearch, or Alexa Fluor 647 Phalloidin, Invitrogen A22287) in blocking buffer overnight at 4°C. After washing with 0.1% Triton X-100 in PBS for 20 min at RT three times, the specimens were mounted on the glass slides for imaging. Volumes of 84.2×84.2×5 μm^3^ were imaged by confocal using LMS880, Zeiss.

### JC-1 staining

The experiments were carried out as previously described with some modifications (Macchi et al., 2013). Fly thoraxes were dissected into halves in Schneider's medium containing 1% cyclodextrin and stained with 8 μM JC-1 (Thermo Fisher Scientific) for 30 min. After washing with Schneider's medium for 5 min two times, the specimens were mounted in Schneider's medium and imaged by confocal microscopy (LMS880, Zeiss). Triplicates of volumes of 84.2×84.2×5 μm^3^ were analyzed. The red and green fluorescent signals from JC1 were measured, and the ratios were calculated using Imaris image analysis software (Bitplane).

### LysoTracker staining

The experiments were carried out as previously described with some modifications (Macchi et al., 2013). Fly thoraxes were dissected into halves in Schneider's medium containing 1% cyclodextrin and stained with 1 μM LysoTracker Red DND-99 (Thermo Fisher Scientific) for 5 min. After washing with Schneider's medium for 5 min two times, the specimens were mounted in Schneider's medium and imaged by confocal microscopy (LMS880, Zeiss). Triplicates of volumes of 84.2×84.2×5 μm^3^ were analyzed. The red fluorescent signals from LysoTracker were measured, and the corresponding volumes were calculated using Imaris image analysis software (Bitplane).

### TUNEL staining

The experiments were carried out as previously described with some modifications (Macchi et al., 2013). Fly thoraxes were dissected into halves in fixation buffer containing 4% paraformaldehyde and 1% Triton X-100 in PBS and fixed for 20 min at RT without shaking. The specimens were washed with 0.1% Triton X-100 in PBS for 20 min at RT three times. After blocking with 5% normal goat serum (Jackson ImmunoResearch) in 0.1% Triton X-100 in PBS for 2 h at RT, the specimens were washed and stained with *in situ* cell death detection kit (Roche) reagents at 37°C for 1 h. The positive control specimens were first incubated with DNase I (2000 U/ml) for 10 min at RT. The negative control specimens were incubated without enzyme terminal transferase. Specimens were washed and stained with primary mouse anti-dsDNA (1:1000, Abcam 27156) in blocking buffer overnight at 4°C. After washing with 0.1% Triton X-100 in PBS for 20 min at RT three times, the specimens were s stained with secondary anti-mouse IgG Alexa-594 (1:500, Jackson ImmunoResearch) or Alexa Fluor 647 Phalloidin (1:1000, Invitrogen A22287) in blocking buffer overnight at 4°C. After washing with 0.1% Triton X-100 in PBS for 20 min at RT three times, the specimens were mounted on glass slides for confocal imaging (LMS880, Zeiss). Volumes of 84.2×84.2×5 μm^3^ were analyzed.

### Apex2 staining electron microscopy (EM) of fly tissue

The protocol was performed as previously described (Hung et al., 2016) with slight modifications (Jiang et al., 2020). Vibratome sections of the fly tissues were fixed in 2% glutaraldehyde in 0.1 M sodium cacodylate with 2 mM CaCl_2_, pH 7. Residual glutaraldehyde was washed off with buffer (2 min, five times) and quenched with 20 mM glycine followed by another wash (2 min, five times). The specimens were subsequently stained with SIGMA *FAST*^™^ DAB (3,3′-Diaminobenzidine tetrahydrochloride) with Metal Enhancer Tablets (Sigma-Aldrich) for 20 min, washed in buffers (10 min, five times) and stained with 1% osmium tetroxide for 30 min. After washing with ddH2O (10 min, three times), the specimens were stained with 1% uranyl acetate overnight. The specimens were further dehydrated and embedded in resin for thin-section and TEM observation.

The TFAM-Apex2 staining signals were analyzed using Amira-Avizo 3D visualization and analysis software (Thermo Fisher Scientific). In short, the EM images were subject to threshold adjustment to select positive Apex2 staining signals. The positive signals were processed using the despeckle and closing functions of Amira-Avizo software. The areas of positive Apex2 staining signals were exported in excel format for analysis. The statistics showed in Fig. 7E included 44, 204, 167, and 94 mitochondria of the control, *CG5903/MIC26-MIC27*-, *Mitofilin/MIC60*-, and *QIL1/MIC13*-knockdown, respectively.

### Apex2 staining EM of cell culture

S2 cells were seeded in six-well culture plates at 1×10^6^ cells/ml and grown for another day to 2–4×10^6^ cells/ml. The cells were transfected with individual vectors pMT-V5-HisB-dMIC60-Apex2-Flag, pMT-V5-HisB-dQIL1-Apex2-Flag, and pMT-V5-HisB-dCG5903/MIC26-MIC27-Apex2-Flag, using a calcium phosphate transfection kit (Invitrogen), and the protein expression was induced by CuSO_4_. The cells were harvested 1-day post-induction, fixed with 2% glutaraldehyde, and subjected to the APEX2 staining procedure as described above.

Subcellular and sub-mitochondrial localization of positive Apex2 staining was analyzed by MetaMorph microscopy automation and image analysis software (Molecular Devices). In brief, the EM image was inverted and adjusted to a consistent threshold to isolate positive Apex2 signals of the target mitochondrion. The intensity in the cristae junction was determined by manual selection of the structure with a circle of 20 pixels in diameter. The intensity in the cristae was also determined based on the manual selection of the structures. The intensity in the IBM was defined by subtracting the intensity in the cristae and cristae junctions from the total intensity. The distribution ratios were calculated relative to total intensity. The analysis showed in Fig. 8E included 11, 17, and 15 mitochondria of *CG5903/MIC26-MIC27*-, *Mitofilin/MIC60*-, and *QIL1/MIC13*-knockdown in the analysis, respectively.

### Quantitative PCR

Fly DNA was extracted by homogenizing about 50 flies in 200 μl of buffer containing 10 mM Tris (pH 8.0), 1 mM EDTA, 25 mM NaCl. Proteinase K (0.2 mg∕ml) was added to the lysates and incubated at 45°C for 30 min followed by inactivation at 95°C for 5 min. The supernatant was collected after centrifugation. Mitochondrial DNA content was analyzed by qPCR using LightCycler^®^ 480 SYBR Green I Master and LightCycler^®^ 480 instrument (Roche). Specific primers for mtDNA (*COIII* Forward: 5^′^-CACGAGAAGGAAC ATACC-3^′^; Reverse: 5^′^-GCGGGTGATAAACTTC TG-3^′^) and nuclear DNA (*RpL32* Forward: 5^′^-GCCGCTTCAAGGGACAGTATCTG −3^′^; Reverse: 5^′^-AAACGCGGTTCT GCATGAG −3^′^) were used. The relative mtDNA *COIII* copy number was normalized to the nuclear *RpL32* copy number. Three independent runs were performed; each run included triplicates with a standard deviation of Cp value less than 0.5.

For RT-qPCR, fly RNA was extracted using RNeasy Mini Kit (Qiagen) and reverse-transcribed using a RevertAid First Strand cDNA Synthesis Kit (Thermo Fisher Scientific). Specific primers for *CG5903/MIC26-MIC27* (Forward: 5^′^-CGGTCTGGCTG GTTTCATCT; Reverse: 5^′^-GGCACTACGGGAACATCCTC-3^′^), *MIC60* (Forward: 5^′^-GATAAGCTGCTGCGCTTGCAGCTCAAAAAG-3^′^; Reverse: 5^′^-GCCACCTTGG CAATGGCATTGATCTCGTTC-3^′^), *QIL1* (Forward: 5^′^-TTTCATCCACATGCTG CCCT-3^′^; Reverse: 5^′^-GCGAGCGGATCGAGGAATAA-3^′^), and *TFAM* (Forward: 5^′^-AACAAAGTCAGGCCCCTAGC-3^′^; Reverse: 5^′^-CTCGACGGTGGTAATCTG GG-3^′^) were used. The relative copies of mtDNA *COIII* transcripts or transcripts from individual genes were normalized to the nuclear *RpL32* transcript copy number. Three independent runs were performed; each run included triplicates with a standard deviation of Cp value less than 0.5.

### Western blot analysis

The fly thoraxes were homogenized in RIPA buffer containing protease inhibitors (cOmplete^TM^, Roche) using a Dounce tissue grinder. Cellular debris was removed by centrifugation at 14,000 ×***g*** for 20 min, 4°C. The supernatants were collected and the protein concentrations were determined by Pierce protein assay (Pierce 660 nm Protein Assay Reagent, Thermo Fisher Scientific). Proteins were loaded at 20 μg/well for SDS-PAGE and western blot analysis.

Mouse anti-Flag M2 (1 μg/ml, Sigma-Aldrich F3165), rabbit anti-alpha tubulin (10000x, Abcam ab18251), anti-mouse IgG-HRP (2000×, Invitrogen 62-6520), and anti-rabbit IgG-HRP (5000x, Abcam ab97051) were used in the study. For quantification of band intensities, ratios of the densitometric signal of individual proteins to that of alpha-tubulin were calculated. The ratios were then normalized to the control samples.

### Climbing assay

The flies were transferred to new culture tubes one day before the analysis. On the day of analysis, flies were transferred to a 100 ml graduated cylinder and knocked down to the bottom of the cylinder when starting video-typing them climbing up to the 100 ml marker of a cylinder (about 18 cm in height). Numbers of flies climbing up to the target line every 10 s in 120 s were calculated and plotted. Numbers of 56, 69, 79, and 62 of the control, *CG5903/MIC26-MIC27*-, *Mitofilin/MIC60*-, and *QIL1/MIC13*-knockdown flies were used in the triplicate analysis, respectively.

## Supplementary Material

Supplementary information
