# Supplementary material for: Drosophila MICOS knockdown impairs mitochondrial structure and function and promotes mitophagy in muscle tissue
Source: Biol Open. 2020 Dec 3;9(12):bio054262. doi: 10.1242/bio.054262 (PMC7725604; doi:10.1242/bio.054262)
Supplement: Supplementary information [file biolopen-9-054262-s1.pdf]

Supplementary figures

Fig S1

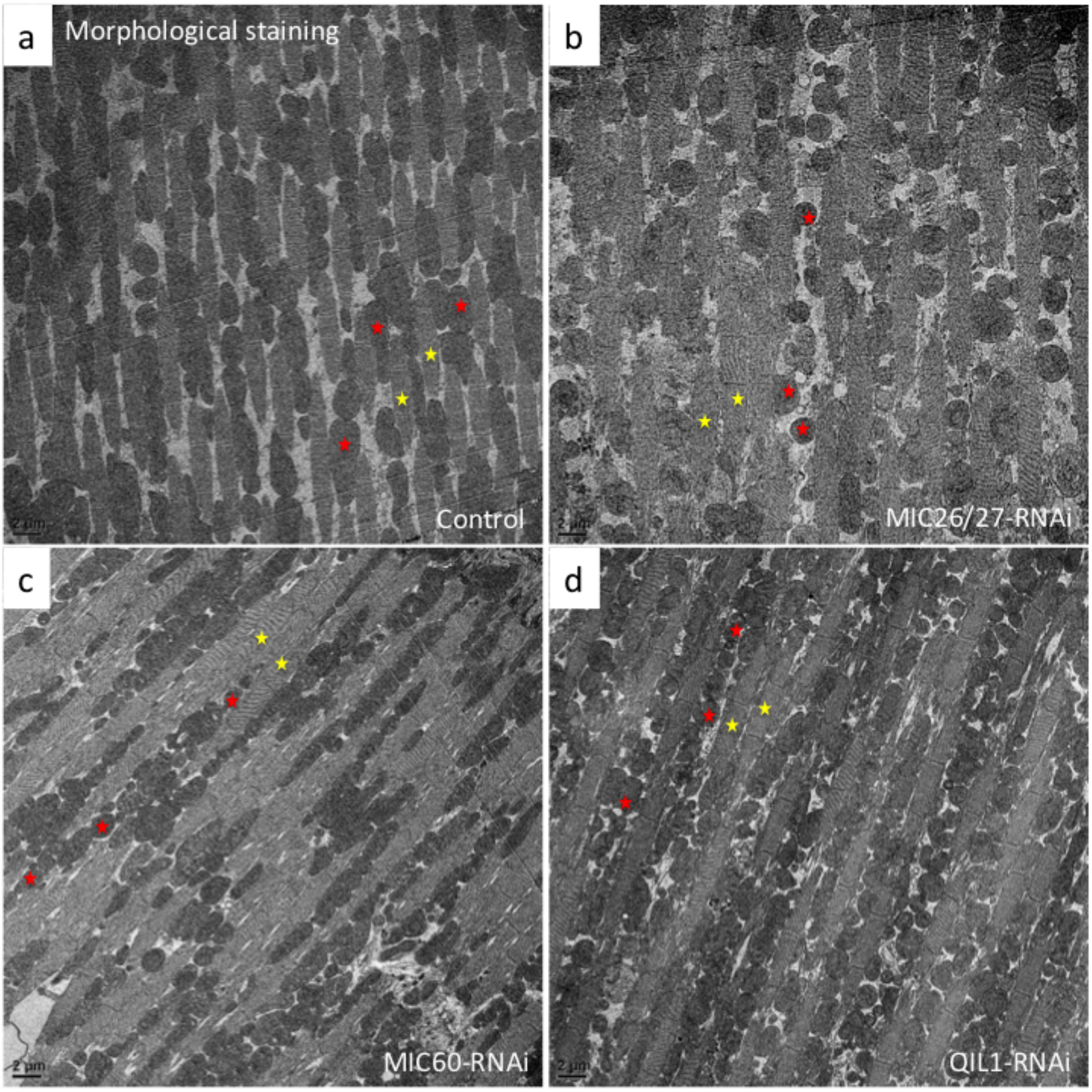

Fig S1. *CG5903/MIC26-MIC27-*, *Mitofilin/MIC60-* and *QIL1/MIC13*-knockdown flies have small mitochondria

(a-d) Thin-section EM images of *Drosophila* IFM from control, *CG5903/MIC26-MIC27-*, *Mitofilin/MIC60-* and *QIL1/MIC13*-knockdown flies. (Red star: mitochondrion; yellow star: muscle fiber.) The flies of *[w, Actin88F-GAL4, CG5903/MIC26-MIC27- RNAi], [w, Actin88F-GAL4, Milton/MIC60- RNAi], and [w, Actin88F-GAL4; QIL1/MIC13- RNAi]* were used.

Biology Open • Supplementary information

Fig S2

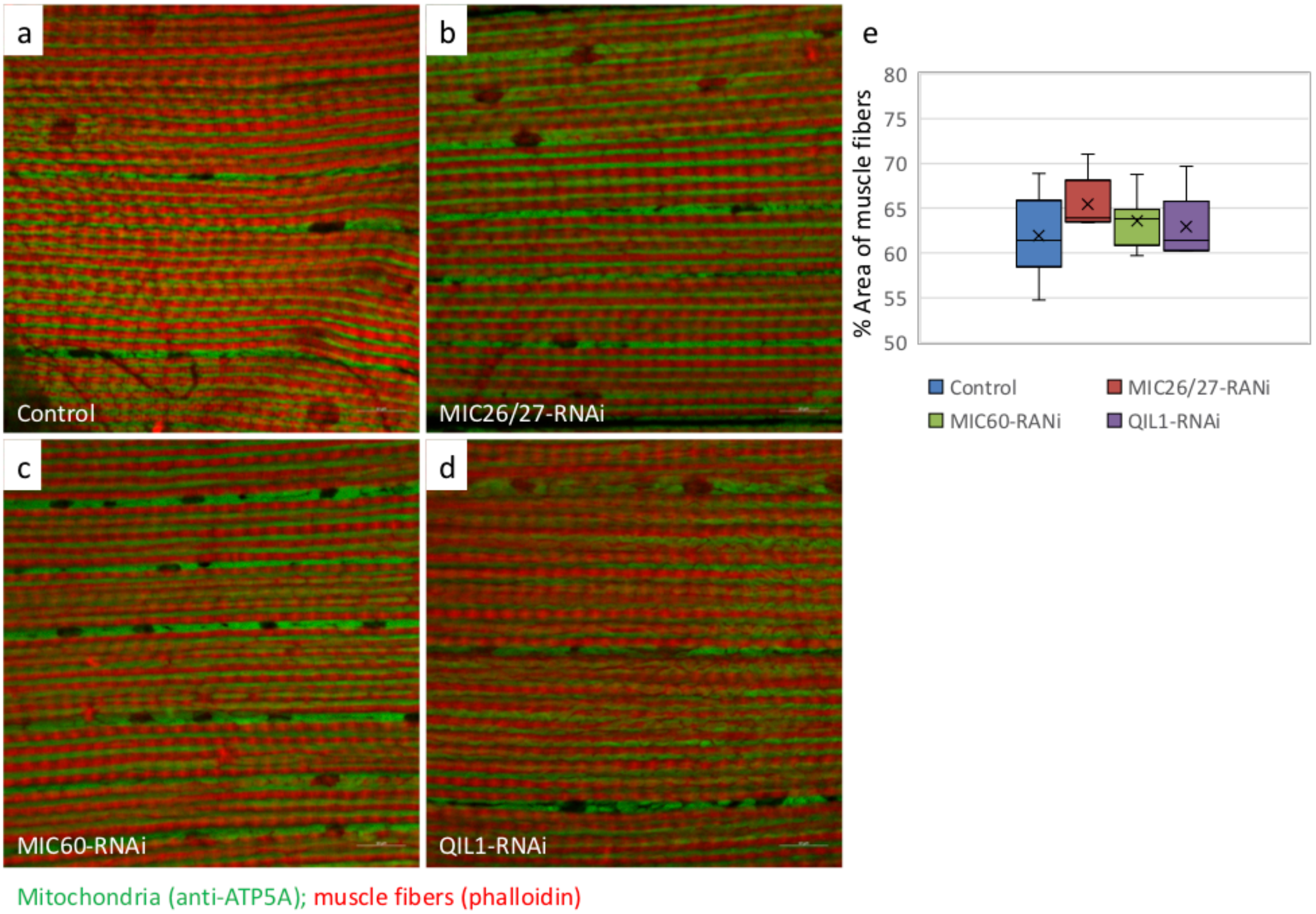

Fig S2. ***CG5903/MIC26-MIC27-*, *Mitofilin/MIC60-*, and *QIL1/MIC13*-knockdown IFMs have normal muscle fiber morphology**

IFM of *CG5903/MIC26-MIC27-*, *Mitofilin/MIC60-*, and *QIL1/MIC13*-knockdown flies were stained for phalloidin (muscle fibers in red) and anti-ATP5A (mitochondria in green) showing intact morphology of muscle tissue. (Volumes of 84.2 x 84.2 x 5 μm^3 were imaged). The flies of *[w, Actin88F-GAL4, CG5903/MIC26-MIC27- RNAi], [w, Actin88F-GAL4, Milton/MIC60- RNAi], and [w, Actin88F-GAL4; QIL1/MIC13- RNAi]* were used.

Biology Open • Supplementary information

Fig S3

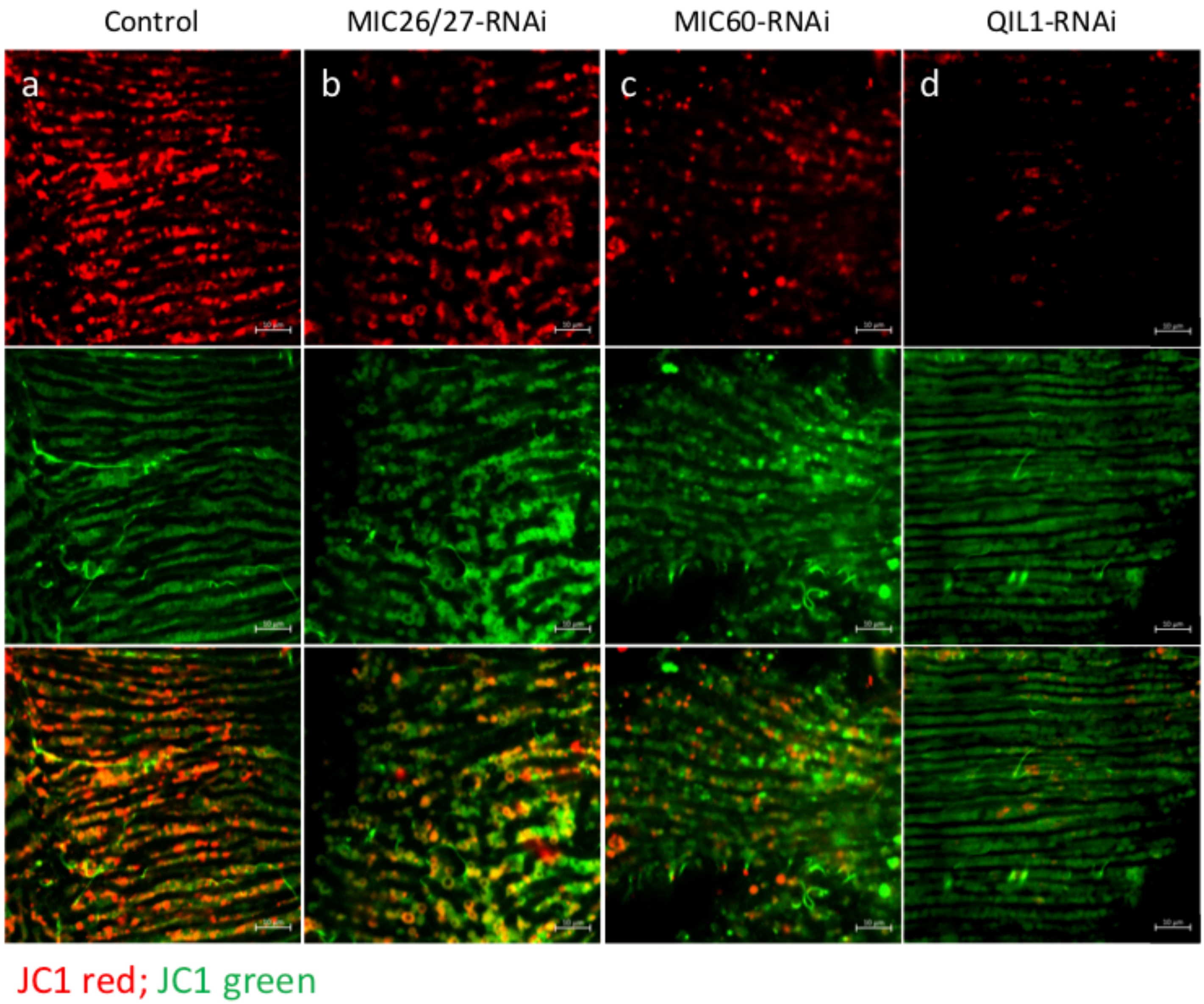

Fig S3. *CG5903/MIC26-MIC27-*, *Mitofilin/MIC60-* and *QIL1/MIC13*-knockdown flies do not exhibit elevated apoptosis

TUNEL staining of *Drosophila* IFM from control, *CG5903/MIC26-MIC27-*, *Mitofilin/MIC60-* and *QIL1/MIC13*-knockdown flies. The positive and negative controls for the TUNEL assay using the control flies were shown. (Volumes of 84.2 x 84.2 x 5 μm^3 were imaged). (Positive TUNEL signals were shown in green; nuclei were stained with anti-dsDNA, red; muscle fibers were stained by phalloidin, purple). The flies of *[w, Actin88F-GAL4, CG5903/MIC26-MIC27- RNAi]*, *[w, Actin88F-GAL4, Milton/MIC60- RNAi], and [w, Actin88F-GAL4; QIL1/MIC13- RNAi]* were used.

Biology Open • Supplementary information

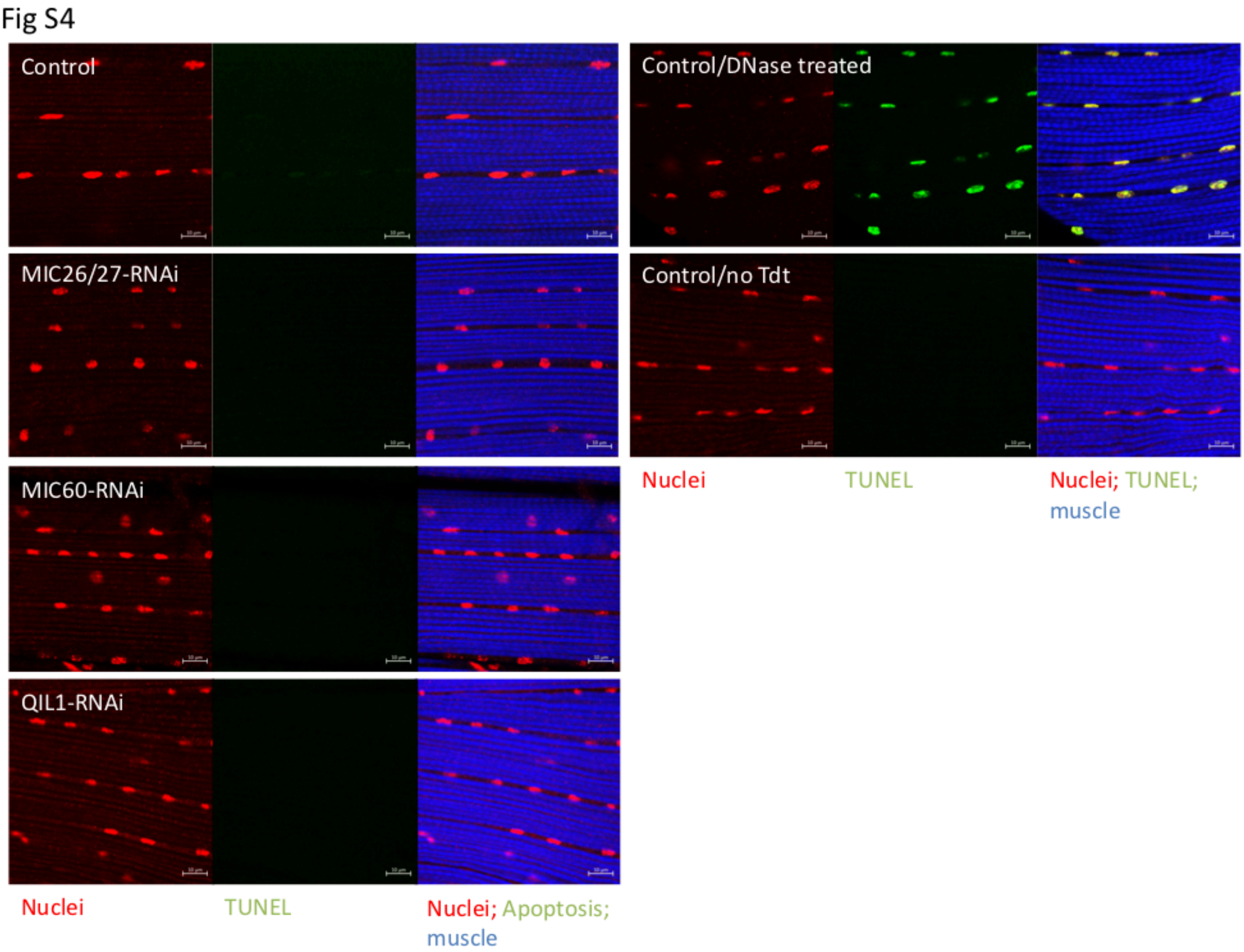

Fig S4. ***CG5903/MIC26-MIC27-*, *Mitofilin/MIC60-* and *QIL1/MIC13*-knockdown flies exhibit low mitochondrial membrane potential**

(a-d) JC1 staining of IFM from *CG5903/MIC26-MIC27-*, *Mitofilin/MIC60-*, and *QIL1/MIC13*-knockdown flies. Red fluorescence marks high mitochondrial membrane potential, whereas green fluorescence indicates low mitochondrial membrane potential. (Volumes of 84.2 x 84.2 x 5 µm^3 were imaged). The flies of *[w, Actin88F-GAL4, CG5903/MIC26-MIC27- RNAi], [w, Actin88F-GAL4, Milton/MIC60- RNAi], and [w, Actin88F-GAL4; QIL1/MIC13- RNAi]* were used.

Biology Open • Supplementary information

Fig S5

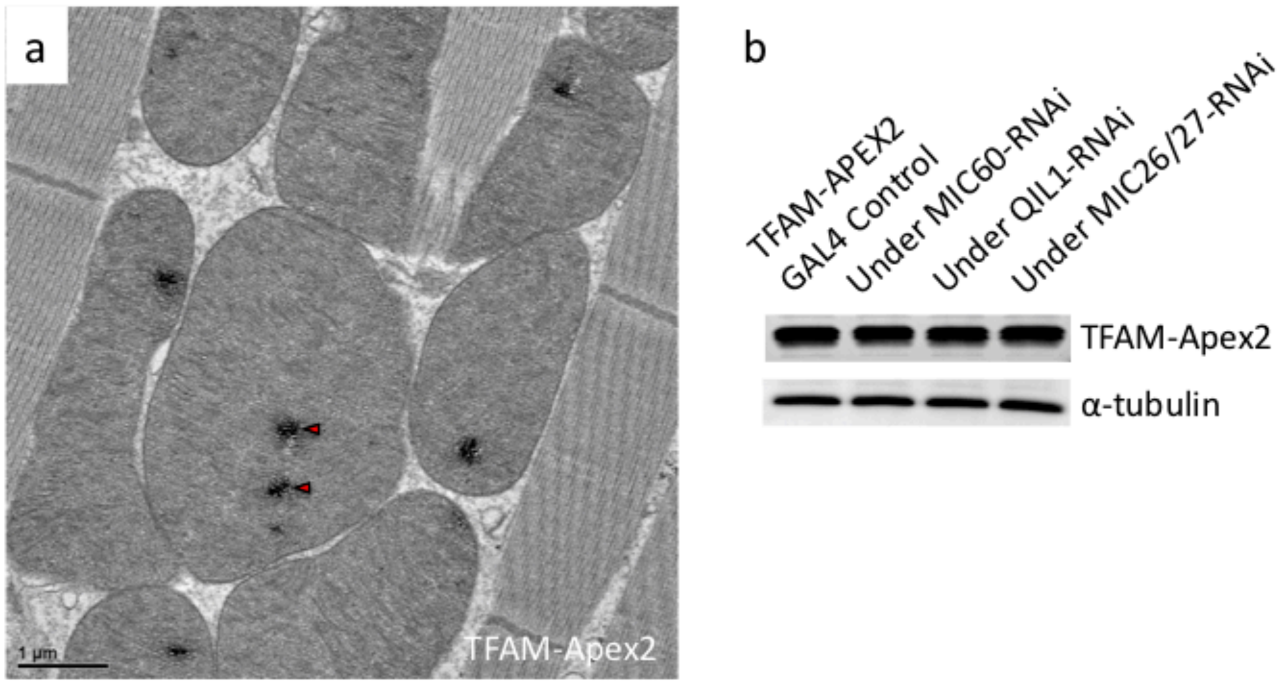

Fig S5. Analysis of TFAM-Apex2 flies

Apex2 EM staining of TFAM-Apex2 knock-in fly showing the mitochondrial nucleoid structure. (b) Western blot analysis of TFAM protein levels in flies with TFAM-Apex2 from GAL4 controls, *CG5903/MIC26-MIC27-*, *Mitofilin/MIC60-* and *QIL1/MIC13-* knockdown. (Red arrowhead: the positive signal of TFAM-Apex2 staining). The flies of *[w, Actin88F-GAL4, CG5903/MIC26-MIC27- RNAi], [w, Actin88F-GAL4, Milton/MIC60- RNAi], and [w, Actin88F-GAL4; QIL1/MIC13- RNAi]* were used.

Biology Open • Supplementary information
